# General Medicine and Therapeutics

**Published:** 1894-04

**Authors:** 


### GENERAL MEDICINE AND THERAPEUTICS.

EDITED BY DR. LUTHER B. GRANDY.

### VACCINATION.

The most conclusive evidence of the protective power of vaccination is illustrated by the results in Germany, where since 1874 all infants are vaccinated, and all school children at the age of twelve years are revaccinated, thereby giving that country an immunity from smallpox greatly above all the contiguous nations.

The effects of vaccination upon the death rate of those attacked by smallpox is conclusively shown by the reports upon the epidemic in Sheffield, England, in 1887–88, where the immunity from the disease was twenty times as great in the vaccinated, and four hundred and eighty times as great from death.

Considering the sore frequently resulting, and the constitutional disturbance so common to it, we believe that the operation of vaccination is a matter of more importance, and requires more attention during its development and progress than has habitually been given to it. This has been especially made apparent during the present season.

It cannot be questioned that the medical profession are quite unanimous in the opinion that the bovine is the only virus to be used in making vaccinations, nor that they regard it of the greatest importance that great care should be used in procuring healthful animals from which it should be obtained, and that the virus should be aseptically taken and dispensed. The opinion recently expressed by Dr. Francis C. Martin, of Roxbury, that only young heifers should be used for obtaining the virus is worthy of most careful consideration.

That a given vaccination shall be protective it should be kept in mind that it must pass through the regular processes of development, forming the papule, the vesicle, and then the vesicle having formed about it a yellowish margin, and on the eighth day becoming umbilicated, at which time there should be found about it a distinct areola. Should the vesicle pustulate and rupture before the eighth day, there has been an imperfect development of the vesicle, and the vaccination would probably prove unprotective.

There can be no doubt that vaccination is too frequently made without proper care for cleanliness of the person at the time, or proper directions as to the care of the sore and constitutional disturbances during the progress of the disease,—*for vaccina is a disease.* The part should be made clean before the virus is inserted; when it becomes inflamed and pustulates it should be carefully protected from the unclean and rough clothing. If the part, the arm for instance, is swollen and painful, it should be suspended in a sling for support. If the patient has a rise in temperature to any marked degree, he should have quiet, a proper diet, and in some instances medication, as in the exanthematous diseases.

In regard to the frequency with which vaccination should be repeated no positive rule can be given; but when it has been made in early childhood, it would seem advisable to revaccinate after puberty. If but few years have elapsed after a successful vaccination, and one should be exposed to smallpox, a revaccination would be the part of due care. The scar, if it contains several well marked pits, indicates that the vaccination is still protective, but this should not be relied upon on exposure to smallpox as giving positive immunity from contracting the disease. Indeed, an attack of the disease itself does not give positive immunity from a subsequent attack.—*Mass. Med. Jour.*

### PERTINENT FACTS ON VACCINE VIRUS.

Keep in a glass-stopper jar in an unheated room in winter and in a refrigerator in summer.

Do not carry in an inside pocket.

Do not use too much water.

Do not abraid too large a surface.

Do not use any dressing.

Do not report until the eighth, or better, the tenth day.

Our plan of operating is as follows:

We scarify by scraping off the scarf-skin from a surface about one-fourth of an inch square over the insertion of the deltoid muscle, scratching the surface thus abraided in two directions.

We touch one side of the ivory point with a drop of water, immediately shaking the excess off; rub this side briskly on the abrasion and then form a paste by using the dry side of the point.

We finish the operation by gently scratching the virus in with the point we have used.

We are then certain of getting some of the vaccine corpuscles within reach of the mouths of the absorbents.

Too much water, with the serum that exudes from the arm, frequently washes away the corpuscles, while too thick a paste, if not pricked in, will dry as a varnish, beyond the reach of the absorbents which have been closed by the rubbing process.

If you protect with a shield, see that it is properly ventilated.

Failures result from

Using unhealthy or poorly fed cattle.

Pressure used in removing lymph.

The clamor of physicians for colorless points.

Hot mail-cars, drug stores and physicians' offices.

Improper methods of operating.

Old goods, or mucilage and croton-oil points, sold at cut-rate prices, while human lives are sacrificed.—*Dr. H. M. Alexander.*

